# Nondetectable Prostate Carcinoma (pT0) after Radical Prostatectomy: A Narrative Review

**DOI:** 10.3390/curroncol29030111

**Published:** 2022-02-23

**Authors:** Nikolaos Kalampokis, Nikolaos Grivas, Markos Karavitakis, Ioannis Leotsakos, Ioannis Katafigiotis, Marcio Covas Moschovas, Henk van der Poel, European Association of Urology (EAU) Young Academic Urologists (YAU) Robotic Urology Working Group

**Affiliations:** 1 Department of Urology, G. Hatzikosta General Hospital, 45001 Ioannina, Greece; kalampokas88@gmail.com; 2 Department of Urology, The Netherlands Cancer Institute-Antoni van Leeuwenhoek Hospital, 1066 CX Amsterdam, The Netherlands; h.vd.poel@nki.nl; 3 Department of Urology, Lefkos Stavros Hospital, 11528 Athens, Greece; markoskaravitakis@yahoo.gr (M.K.); ioannisdleotsakos@gmail.com (I.L.); katafigiotis.giannis@gmail.com (I.K.); 4 Department of Urology, University General Hospital of Heraklion, University of Crete, Medical School, 14122 Heraklion, Greece; 5 Department of Urology, Medical School, National & Kapodistrian University of Athens, 14122 Athens, Greece; 6 Department of Urology, Advent Health Global Robotics Institute, Celebration, FL 34747, USA; marcio.moschovas.md@adventhealth.com

**Keywords:** prostate cancer, prostatectomy, vanishing cancer

## Abstract

(1) Background: Following radical prostatectomy (RP), the absence of a demonstrable tumor on the specimen of a previously histologically proven malignancy is known as the pT0 stage. The aim of our present study is to perform a narrative review of current literature in order to determine the frequency and oncological outcomes in patients with pT0 disease. (2) Methods: A narrative review of all available literature was performed. (3) Results: The incidence of pT0 ranges between 0.07% and 1.3%. Predictors of the pT0 stage are only a single biopsy core with low-grade cancer, a cancer length not exceeding 2 mm and a high prostate volume. Biochemical recurrence ranges between 0 and 11%. (4) Conclusions: The absence of malignancy in the RP specimen despite a previous positive biopsy is a rare and unpredictable finding. Although the prognosis is considered to be excellent in most of the cases, a continued close follow-up is warranted.

## 1. Introduction

Over the past decades, the implementation of a widely accepted screening program for the early detection of prostate cancer has resulted in even more patients being diagnosed with low-grade, small in size malignancies. Following radical prostatectomy (RP), the absence of a demonstrable tumor on the specimen of a previously histologically proven malignancy is known as the pT0 stage. Although this is a well-known phenomenon for individuals receiving neoadjuvant hormonal therapy (NHT), the incidence of pT0 among patients who are directly treated with RP without prior androgen deprivation therapy (ADT) is <2% [1,2,3,4].

The aim of our present study is to perform a review of current literature in order to determine the frequency and oncological outcomes in patients with pT0 disease, as well as possible factors serving as predictors of the pT0 stage in candidates for RP.

## 2. Materials and Methods

**Figure 1:**
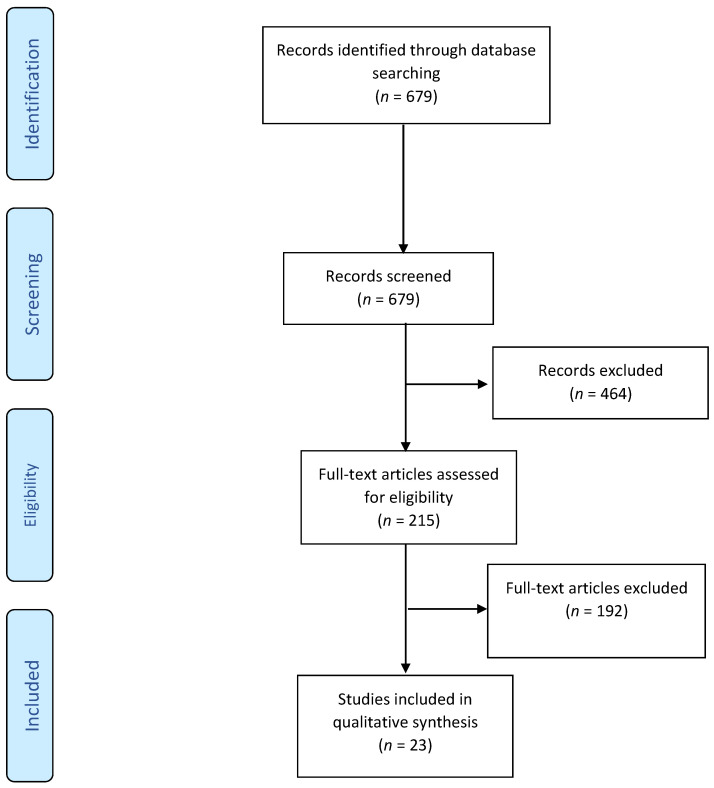
Preferred Reporting Items for Systematic Reviews and Meta-analysis.

Medline Epub Ahead of Print, In-Process and Other Non-Indexed Citations, Ovid MEDLINE(R) Daily and Ovid MEDLINE(R) 1946 to 30 November 2021 were systematically searched to detect all relevant studies based on the following literature search strategy: (undetectable OR pT0 OR vanishing) AND (prostatectomy). After excluding citations in abstract form, and non-English citations, titles/abstracts of full papers were screened. Review articles, editorial letters and comments were excluded. Two review authors (NK and NG) independently scanned the titles, abstracts or both of every record retrieved, to determine which studies should be further assessed and extracted all data. Disagreements were resolved through consensus or after consultation with a third review author (MK). A total of 679 unique abstracts were identified by the search and 215 were selected for full-text screening. After full-text screening, 23 studies met the inclusion criteria (Figure 1).

## 3. Results

### 3.1. Frequency and Possible Causes of Cancer Absence in Prostatectomy Specimen

The first cases of pT0 disease were reported by Goldstein et al. back in 1995 and they were described as the vanishing cancer phenomenon [2]. The authors re-evaluated the data of 13 patients (11 with minimal and two with no cancer in the prostatectomy specimen) and they concluded that even after meticulous histopathologic examination, cancer may be impossible to be found in every RP specimen.

In three consecutive studies, a group of pathologists reported their experience with patients diagnosed and treated for prostate cancer in the Johns Hopkins Hospital over a period of 9 years (1997–2005) [4,5,6]. According to them, there was an increase in the number of patients diagnosed with pT0 by almost five times (from 0.07% in 1997 to 0.34% in 2005).

In 2004, Bostwick et al. found that 38 out of 6843 patients, who were treated with RP at their institution during a 30-year period, had no sign of malignancy in the surgical specimen [3]. Interestingly, they reported a decrease of the vanishing cancer incidence by more than 10 times, while at the time of publication, the current incidence was estimated at approximately 0.2%.

More recently, a pooled analysis by Gross et al. included more than 18,000 patients and reported a pT0 rate equal to 0.4% (CI: 0.3–0.5%) [7]. Similarly, in 2019, Knipper et al. performed a large population-based analysis using the SEER database and reported a pT0 rate of 0.2% [8].

As regards the association of the pT0 stage with specific racial characteristics, three large studies conducted in French and German institutions reported an incidence ranging from 0.4% to 0.8% [9,10,11], while a study of 702 Asian patients showed a rate of pT0 staging equal to 1.3% [12].

So far, several mechanisms have been proposed in an effort to explain the absence of detectable malignancy in the RP specimen. According to Descazeaud et al. the most plausible explanation would be that of a high-volume prostate, which would make it difficult for a pathologist to detect small in size tumors [9]. Another possible explanation would be that specimen mix-up and several techniques have been established so far with an aim to avoid such a case of malpractice [4]. Other explanations would be (1) the initial core biopsy was positive for an entity mimicking prostate cancer (e.g., high-grade prostate intraepithelial neoplasia); (2) the tumor was entirely removed during a transurethral resection (TURP); or (3) pre-operative ADT resulted in downstaging of the disease [13].

### 3.2. Possible Pre-Operative Predictors of pT0 Stage

So far, there have been several studies trying to confirm the existence of pre-operative factors that could help us predict which of the patients would be more likely to receive the diagnosis of pT0 disease following RP.

A large single-institution study by Descazeaud et al. was probably the first one trying to create a predictive model for the pT0 stage [9]. According to the authors, the simultaneous existence of only one biopsy core with low-grade cancer, a cancer length on biopsy not exceeding 2 mm and a prostate larger than 60 g in weight was found to have a specificity of 99% and a sensitivity of 82% in predicting pT0 on radical prostatectomy. Interestingly, the negative predictive value of their model was found to be equal to 99%, which means that it would be almost impossible for a patient not sharing all the aforementioned characteristics to be diagnosed with pT0.

Working towards the same goal, Bream at al. examined a North American population and concluded that patients with co-existence of a PSA level below 7.5 ng/mL, a Gleason score of 6, a clinical T1c stage and a single biopsy core with cancer occupying less than 1% of tissue could be probably better served with active surveillance instead of RP, unless a repeat biopsy yields more concerning findings [14].

In 2011, Capitanio et al. conducted a study, which included patients diagnosed with T1a and T1b disease after being operated on for benign prostatic hyperplasia, and according to them pT0 cancer was, as expected, associated with lower prostate specific antigen (PSA) levels [15]. Similarly, Moreira et al. after examining patients regardless of pre-operative treatment, showed that a lower Gleason score and PSA levels as well as any pre-operative treatment in the form of ADT or radiotherapy, were found to be independent predictors of the pT0 stage with an accuracy equal to 75% [16].

In 2018, Chung et al. conducted a study, which included patients undergoing RP after being diagnosed with incidental prostate cancer (T1a–1b) [17]. Among the 95 patients of the study, there were 28 individuals with absence of malignancy in the prostatectomy specimen (pT0). It is worth mentioning that according to their findings, patients with incidental cancer who have both an invisible lesion on multiparametric magnetic resonance imaging (MRI) and PSA density lower than 0.08 following TURP could be safely considered for active surveillance instead of radical prostatectomy.

Finally, a SEER-based analysis by Knipper et al. produced a model with only three variables reaching independent predictor status, namely the number of positive biopsy cores, the number of biopsies taken and the Gleason score [8]. Nevertheless, according to them, the extremely low prevalence of the under examination clinical entity (0.2% according to the authors) could not guarantee that a model with accuracy equal to 79% would be of any usefulness in everyday clinical practice.

Data from these older studies show that the vast majority of men with pT0 had low-risk PCa which, today, should be offered active surveillance. Risk stratification of patients is of the utmost importance in order to avoid over-treatment and its possible side effects.

### 3.3. pT0 Stage following Hormonal Therapy

Among others, several studies have dealt with the correlation between NHT and pT0 following RP. The main goal of neoadjuvant is to reduce positive surgical margins and rates of disease recurrence. Hormonal pre-treatment is already known to cause a reduction of the tumor size [18] by causing a variety of regressive changes, thus leaving scattered malignant cells behind [19,20] and making the post-treatment detection of the tumor extremely difficult. Nevertheless, it has been shown that even if the initial pathologic evaluation failed to detect the presence of tumors, an extensive re-evaluation would identify malignant cells in more than 60% of those cases.

In 2000, Kollermann et al. compared the effect of PSA-monitored prolonged neoadjuvant endocrine treatment (PPNET) on the number of post-operative pT0 reports, when compared to the standard 3-month treatment schedule [21]. According to their findings, a patient receiving prolonged hormonal treatment (mean duration = 9 months) was three times more likely to receive a diagnosis of the pT0 stage, which indirectly implies that the 3-month schedule does not exploit the full potential of neoadjuvant treatment.

### 3.4. pT0 Diagnosis: Follow-Up and Oncological Outcomes

A summary of oncological outcomes of patients with the pT0 stage are presented in Table 1. In one of the first studies dealing with the prognosis of the pT0 stage, a research team from Berlin analyzed a group of 174 patients receiving pre-operative ADT and found that 21% of them were staged as pT0 after RP [13]. When the aforementioned patients were matched for a Gleason score with patients diagnosed as pT2–3, there was no difference in PSA free survival rate, which according to the authors means that biochemical progression does occur despite possible downstaging to pT0 after prolonged NHT.

In 2003, Herkommer et al. presented a study on the incidence of pT0 on a nation-wide basis (Germany). Among 3609 patients undergoing RP, there were 28 individuals who were staged as pT0 (0.8%) [10]. All patients, irrespective of stage, had undetectable PSA levels within 4 weeks after operation. Moreover, none of them had biochemical or clinical progression of their disease during follow-up (mean period: 62 months). During the same year, an article published by Kollermann et al. tried to shed some light on the hypothesis that in pT0 cases following prolonged NHT, the biochemical relapse is not only extremely rare but also derives from systemic disease recurrence [22]. Based on their findings, both hypotheses were disproved. In total, 18.4% (7 out of 38) of the pT0 patients had a median time to PSA relapse equal to 14 months, while localization studies showed at least a local source of PSA production for six out of seven patients. More specifically, in half of the cases local recurrence was malignant in nature.

In 2004, in a large single-institution retrospective study collecting data over a period of 30 years, none of the 38 patients with the pT0 stage had either biochemical or clinical recurrence over a mean follow-up period of 9.6 years [3]. Similarly, a smaller study showed no recurrence but the sample size was too small (11 patients with pT0) and the mean follow-up period was only 30 months [9].

In 2009, Bessede et al. released their findings of a multi-center study on 7693 patients who underwent radical prostatectomy without hormonal pretreatment [11]. They found 30 cases of the pT0 stage, which were separated into nonsignificant, intermediate and significant risk subgroups based on pre-operative clinical and histopathological characteristics. According to the authors, none of those patients had a disease recurrence at 82-month follow-up, which according to them is translated into an excellent prognosis for pT0 patients irrespective of pre-treatment criteria (i.e., clinical stage, PSA value, Gleason score on biopsy). On the other hand, Gurski et al. concluded that the recurrence rate of their pT0 patients was clinically significant since 26% (six of 23) of them developed biochemical recurrence during follow-up [23]. A possible explanation for their findings could be a long follow-up period, which was described by them as ‘’adequate’’ without giving further details of the exact duration.

More than 20,000 patients who underwent RP between 1987 and 2012 at the Mayo Clinic were included in a retrospective study conducted by Moreira and his colleagues [16]. Seven of the 62 patients (11%) who were diagnosed as pT0 developed recurrence after a median follow-up of 10.9 years. Moreover, when patients of the pT0 group were matched with patients of the non-pT0 group, they were reported to have a statistically significant better recurrence-free survival rate (*p* = 0.008). Interestingly, all of the patients experiencing recurrence had received pre-operative treatment and potential explanations for that finding include the fact that more aggressive tumors are traditionally selected to receive neoadjuvant treatment and the masking effects of previous treatments on cancer cells.

Finally, a large population-based study [8] showed that at a 9-year follow-up the cancer specific survival rate for pT0 patients was equal to 99.5% and almost identical to that of the non-pT0 group (98.8%). However, according to the authors the very low prevalence of the pT0 disease could not guarantee any meaningful statistical comparison.

**Table 1:** Oncological outcomes of patients with pT0 stage.

Study	pT0 Cases (*n*)	Follow-Up Duration	Outcome
Gurski et al. [23]	23	Reported as adequate	26% developed biochemical recurrence
Knipper et al. [8]	358	9 years	3 cancer specific deaths (99.5% cancer-specific survival)
Chung et al. [17]	28	68.37 months (median)	No clinical or biochemical recurrence
Moreira et al. [16]	62	10.9 years (median)	11% with disease recurrence 1.6% with systemic progression
Bream et al. [14]	4	3 months–10 years	No clinical or biochemical recurrence
Bessède et al. [11]	30	82 months (median)	No biochemical recurrence
Trpkov et al. [1]	9	23.8 months (mean)	No clinical or biochemical recurrence
Descazeaud et al. [9]	9	30 months (mean)	No clinical or biochemical recurrence
Köllermann et al. [13]	36	47 months for the pT0 group (median)	19.4% with biochemical recurrence
Bostwick et al. [3]	38	9.6 years (mean)	No clinical recurrence No biochemical recurrence (PSA available only for 32 of 38 patients)
Herkommer et al. [10]	13	62 months(median)	No clinical or biochemical recurrence
Köllermann et al. [22]	38	47 months (median)	18.4% with biochemical recurrence 7.9% with clinical recurrence

## 4. Conclusions

In summary, the absence of malignancy in the RP specimen despite previous positive biopsy is a rare and unpredictable finding, which needs special management because of possible medicolegal repercussions. It is generally associated with features of low-risk cancer and pre-operative hormonal treatment. The findings of our review strengthen the active surveillance strategy in low-risk cases instead of RP. So far, several models serving as pre-operative predictors of the pT0 stage have been proposed, but none of them have gained wide acceptance in everyday clinical practice. Although the prognosis is considered to be excellent in most of the cases, a continued close follow-up is warranted.
